# Interpretable Model for Clinical Use in Left Atrial Appendage Segmentation via an Optimised Deformable‐Attention U‐Net With Spatial–Channel Fusion

**DOI:** 10.1049/htl2.70088

**Published:** 2026-06-04

**Authors:** Ali Pakizeh Moghadam, Javad Haddadnia

**Affiliations:** ^1^ Department of Electrical and Computer Engineering Hakim Sabzevari University Sabzevar Iran

**Keywords:** bidirectional attention blocks, deep learning, deformable attention U‐Net, echocardiography images, hyperparameter optimisation, LAA segmentation

## Abstract

Accurate segmentation of the left atrial appendage (LAA) is essential for device occlusion planning in atrial fibrillation patients who cannot receive anticoagulation. Yet 3D echocardiography suffers from low signal‐to‐noise ratio, anisotropy and marked morphological variability, increasing overfitting risk and reliance on operator‐dependent post‐processing. Existing U‐Net variants capture local detail but often miss long‐range dependencies, while transformers improve context at the cost of boundary precision; semi‐automated pipelines still require experts. We present a fully automated, AERO‐optimised DAT‐DAD U‐Net with SE‐augmented skip fusion. Deformable attention transformers (DAT) provide content‐adaptive global context, while a dual attention with deformable convolution (DAD) block refines rims and addresses shape irregularities; spatial‐channel squeeze‐and‐excitation improves multi‐scale fusion. Hyperparameters are selected by AERO, a surrogate‐ and multi‐fidelity‐driven optimiser balancing exploration and exploitation under limited data in practice. Validation used a 22‐patient 3D echocardiography cohort from King's College Hospital. Volumes were reformatted into axial 2D slices, trained with on‐the‐fly anatomy‐preserving augmentation and evaluated using strict patient‐wise splits. The model achieved Dice 0.8925 ± 0.0144, IoU 0.8026 ± 0.0156 and HD95 9.14 ± 1.96 mm. Ablations confirmed additive gains from DAT, DAD and SE, with faster convergence and a lower error floor, supporting operator‐light, time‐sensitive LAA workflows.

## Graphical abstract

Accurate left atrial appendage segmentation on 3D echocardiography is critical for occlusion planning but is hindered by low SNR, anisotropy and limited cohorts; we propose a fully automated, AERO‐optimised DAT–DAD U‐Net with SE‐augmented skip fusion. The method couples deformable attention for content‐adaptive global context with dual attention plus deformable convolution for crisp rims, with hyperparameters selected via a surrogate/multi‐fidelity search. On a 22‐patient King's College Hospital cohort (patient‐wise split), it achieves Dice 0.8925 ± 0.0144, IoU 0.8026 ± 0.0156, HD95 9.14 ± 1.96 mm and 0.54 s/image, supporting time‐sensitive, operator‐light clinical workflows.

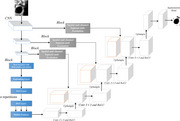

## 1 Introduction

Left atrial appendage (LAA) occlusion has emerged as an effective strategy for stroke prevention in patients with atrial fibrillation, particularly for those unable to tolerate anticoagulant therapy [1, 2]. In this approach, occlusion devices are precisely positioned at the LAA landing zone to block blood flow to the appendage. Correct sizing and positioning of these devices are crucial for procedural success and for minimising post‐procedural complications [3, 4].

Current methods for device sizing and planning largely rely on manual analysis of echocardiographic and computed tomography (CT) images. This process is time‐consuming and costly, demanding high levels of expertise due to anatomical complexity, and is prone to errors in device sizing selection [4, 5]. Semi‐automated and automated segmentation methods for LAA have gained attention in recent years to facilitate the occlusion process. By reducing dependence on clinician expertise and improving accuracy, these methods enhance the efficiency and safety of the procedure. Among these techniques, deep learning and AI‐based image analysis have shown promise in reducing image analysis time and increasing segmentation precision [6].

Nevertheless, one of the main challenges in LAA segmentation is the precise identification of the device landing zone, given the anatomical variations in LAA structure, necessitating algorithms with high adaptability. The development and implementation of AI models could contribute to faster, more accurate decision‐making, thereby improving clinical outcomes [7, 8, 9, 10, 11]. Many studies have investigated automated and semi‐automated LAA segmentation in three‐dimensional echocardiographic images [12, 13, 14, 15]. For instance, Pakizeh Moghadam et al. [1] developed a semi‐automated segmentation algorithm for LAA that demonstrated high accuracy in analysing CT images and identifying an appropriate occlusion zone. This algorithm leveraged image processing techniques and deep learning to extract key LAA features, significantly enhancing occlusion planning precision. Additionally, recent clinical reviews have underscored the increasing importance of accurate anatomical assessment for LAA occlusion planning [2].

Accurate segmentation of the LAA is essential for occlusion planning in atrial fibrillation patients who are contraindicated for anticoagulation, as precise boundary delineation directly affects procedural safety and efficacy. In response to persistent challenges—highly variable LAA anatomy, weak and irregular boundaries and the need for practical deployment—we propose a deployment‐oriented, fully automated framework that unifies architectural advances with principled hyperparameter optimisation to deliver both higher accuracy and operational efficiency for three‐dimensional echocardiographic imaging.

Accordingly, this study introduces a U‐Net–based architecture that combines a Deformable‐Attention Transformer (DAT) in the encoder with a dual‐attention‐with‐deformable (DAD) block and spatial‐channel squeeze‐and‐excitation on the skip connections (DAT–DAD U‐Net). DAT provides content‐adaptive receptive fields and long‐range aggregation for complex LAA geometry, while DAD sharpens local boundary cues; SE‐augmented skips fuse context in a way that is robust to morphology shifts. In addition, deformable convolution is integrated within the attention pathways to better capture inconsistent boundary patterns typical of the LAA landing zone. Together, these components are explicitly tailored to the known failure modes of prior LAA methods, including blurred borders, loss of context and instability across shapes.

To ensure the model is tuned for both accuracy and practical use, we further incorporate an adaptive exploration with surrogate and multi‐fidelity optimiser (AERO), a population‐based, surrogate‐assisted procedure that automatically selects architectural and training hyperparameters under a multi‐objective view (Dice as the primary objective, with latency and peak memory as deployment costs). AERO combines cost‐aware surrogate ranking, adaptive exploration‐exploitation and multi‐fidelity training to find Pareto‐efficient configurations that conventional tuning strategies typically miss.

The study pursues the following objectives: (O1) precise LAA landing‐zone delineation with high boundary fidelity; (O2) robustness across diverse LAA morphologies; (O3) deployment‐aligned efficiency in terms of inference time and memory footprint; and (O4) reduced reliance on manual interpretation. In our experiments, we meet these objectives, achieving strong accuracy (e.g., Dice ≈ 0.893, with HD95 reported in mm) with stable performance across shapes, while maintaining competitive inference time (per‐image) and memory usage.

Key contributions of the research include:

1. A DAT–DAD U‐Net with SE‐augmented skip fusion and deformable convolution within attention paths, purpose‐built to enhance boundary precision and morphological robustness in LAA segmentation.
2. AERO, a novel, deployment‐aware hyperparameter optimisation scheme that couples cost‐aware surrogate modelling with multi‐fidelity training and adaptive exploration–exploitation, yielding Pareto‐optimal accuracy–latency–memory trade‐offs.
3. A small‐cohort training strategy that pairs strictly patient‐wise splits with on‐the‐fly, anatomy‐preserving augmentations (limited in‐plane rotations, isotropic scaling, elastic warps, brightness/contrast shifts and region‐aware crops) whose probabilities and intensities are co‐optimised by AERO. This raises effective sample diversity without inflating the dataset or risking leakage, improving robustness on 3D echocardiography with limited subjects.
4. A patient‐wise setup with comprehensive ablations (DAT, DAD and SE), clear metric definitions (Dice/IoU and HD95 with units) and reporting of resource metrics (inference time and peak memory) to support real‐world adoption, together with an end‐to‐end automated pipeline for LAA landing‐zone segmentation from 3D echocardiography that reduces manual burden and aligns with procedural planning needs.

The remainder of this article is organised as follows: Section 2 reviews existing LAA segmentation methods and their limitations; Section 3 details the proposed DAT–DAD U‐Net and the AERO optimisation framework; Section 4 presents the objective‐aligned experimental results; Section 5 discusses the findings, comparative context and limitations; and Section 6 concludes the paper.

## 2 Related Work

LAA occlusion has become an important strategy for stroke prevention in patients with atrial fibrillation who are unsuitable for long‐term anticoagulation; consequently, accurate LAA delineation and landing‐zone assessment are critical for device sizing, procedural planning and complication reduction. Despite the increasing clinical importance of this topic, the literature remains relatively limited, especially for automated segmentation in echocardiographic imaging, where low contrast, speckle noise and substantial anatomical variability make the task particularly challenging.

Batko et al. [16] investigated the anatomical relationship between the LAA and the left coronary artery to determine safe distances for LAA closure. Using CT images, they showed that distances below 2 mm may present substantial procedural risk. Similarly, Wang et al. [17] studied 96 patients and reported that CT‐based preoperative planning improved procedural efficiency and increased first‐attempt occlusion success. Although such CT‐based approaches provide valuable anatomical detail, they depend on ionising imaging, additional cost and workflow complexity, which may limit their routine applicability in all clinical settings.

Zhu et al. [18] proposed an adversarial latent‐space alignment network for LAA segmentation in echocardiographic images and demonstrated improved boundary accuracy compared with earlier approaches. Pereira et al. [19] showed in a systematic review and meta‐analysis that adding cardiac computed tomography angiography (CCTA) to conventional pre‐procedural planning for LAA occlusion improved device selection accuracy and procedural success, although the approach still relies on advanced imaging infrastructure and CT‐based analysis. Heidari et al. [20] further explored virtual‐reality‐assisted LAA measurements, showing that immersive 3D visualisation may assist clinicians in device selection, but this strategy also depends on specialised hardware and workflow support.

Michiels et al. [6] introduced a fully automated AI‐based framework for CT analysis in LAA closure planning, improving accuracy while substantially reducing image‐processing time. More recent studies have further shown that automated deep‐learning‐based LAA analysis can reduce manual workload and improve measurement consistency. For example, Santos et al. [21] evaluated several U‐Net‐based architectures for automated LAA segmentation and volume quantification from cardiac CT and demonstrated that deep learning can provide reproducible and clinically useful LAA analysis with reduced operator dependence. Selvais et al. [22], Mahmoudi et al. [23] and Stomaci et al. [24] emphasised, from different procedural perspectives, that anatomical complexity, post‐procedural remodelling and patient‐specific device selection remain central challenges in LAA occlusion planning.

Della Rocca et al. [25] examined anatomical changes in the LAA after radiofrequency‐based ostial isolation and reported that marked ostial size reduction may have implications for subsequent occlusion planning. Bertsche et al. [26] investigated contrast‐free cardiac MRI for preprocedural LAA closure planning and showed its potential value for device selection. Sulague et al. [27] reviewed broader LAA applications in atrial fibrillation, while Korsholm et al. [28] demonstrated that LAA closure remains feasible even in patients with prior atrial septal defect closure, provided that planning is sufficiently precise.

Fernandes et al. [29] evaluated deep learning models for LAA segmentation in ultrasound images and showed that automated analysis can support more precise procedural planning. Sarapardeh et al. [30] proposed a neural‐network‐based framework using 3D echocardiography for LAA segmentation and Watchman device sizing, reporting strong agreement with manual analysis, although the study was limited by dataset size. Liu et al. [31] introduced LAFlowNet, which uses point‐cloud learning to estimate blood‐flow and pressure fields in the left atrium and LAA, demonstrating the broader role of AI in patient‐specific LAA analysis. Wu et al. [32] proposed TransFusion, a transformer‐based framework for LAA segmentation in 3D echocardiography, showing that multi‐branch fusion can improve spatial‐semantic representation and segmentation robustness.

More recently, Ghayoumi Zadeh et al. [33] reported an echocardiography‐based LAA segmentation framework that used hierarchical cross‐scale consistency with gated routing to refine skip fusion and improve boundary stability under strict patient‐level evaluation, highlighting the continuing importance of robust multi‐scale feature integration in data‐limited echo settings. In parallel, recent studies on LAA segmentation have increasingly focused on architectures that better balance fine boundary preservation with broader contextual modelling in challenging echocardiographic data. For example, Barbosa et al. [34] performed a comparative study of deep‐learning methods for LAA segmentation in 3D transesophageal echocardiography and showed that modern encoder‐decoder architectures can achieve clinically meaningful delineation accuracy while substantially reducing manual effort. More recently, Zhu et al. [35] proposed a local‐to‐global ConvMamba‐based framework for joint LAA morphology segmentation and thrombus‐related image analysis, demonstrating that explicitly combining local structural sensitivity with global dependency modelling can improve robustness in anatomically complex and noisy LAA images. Together, these studies reinforce the view that accurate LAA segmentation increasingly depends on methods that integrate local‐detail preservation, context‐aware representation learning and robustness to echocardiographic variability.

Nevertheless, important challenges remain insufficiently addressed in LAA segmentation, particularly in echocardiographic imaging. First, the LAA often presents weak, fuzzy and irregular boundaries that make precise delineation difficult. Second, substantial inter‐patient anatomical variability limits the robustness of conventional models. Third, the modest size of most available datasets increases the risk of overfitting and reduces generalisability. Fourth, conventional CNN‐based models are effective at capturing local appearance but often miss long‐range dependencies, whereas Transformer‐based models improve contextual aggregation but may still compromise boundary precision or incur higher computational cost. Finally, from a clinical perspective, segmentation frameworks must not only be accurate but also efficient, reproducible and minimally dependent on expert interaction.

Motivated by these limitations, this study proposes a deployment‐oriented DAT–DAD U‐Net that combines a DAT encoder, a DAD block, and spatial‐channel squeeze‐and‐excitation fusion in the skip pathways. In addition, the proposed framework incorporates AERO‐based hyperparameter optimisation to improve the balance among segmentation accuracy, boundary fidelity and practical deployment efficiency. In this way, the present work is designed not merely to improve overlap scores, but also to more directly address the unresolved challenges identified in prior LAA segmentation and planning studies.

## 3 Proposed Model

The model is based on a U‐Net backbone augmented with a DAT encoder, a DAD module for boundary sharpening and spatial‐channel squeeze‐and‐excitation (SE) units on the skip connections, followed by a decoder that aggregates multi‐scale context. Deformable convolutions are embedded within the attention pathways to adapt the receptive field to irregular LAA morphology. Together, these components improve boundary fidelity and robustness in 3D echocardiography while maintaining efficient inference.

### 3.1 Image Augmentation

For LAA landing‐zone segmentation in occlusion planning, we adopt a conservative, anatomy‐preserving augmentation policy that is consistent with our dataset protocol. Augmentations are applied on‐the‐fly to training data only (never to validation or test) under patient‐wise splits, so that diversity is increased without introducing leakage or inflating the nominal sample count. Because 3D volumes are trained as axial 2D slices and evaluated case‐wise, all transforms are designed to preserve cardiac laterality and the geometric plausibility of the LAA.

**FIGURE 1:**
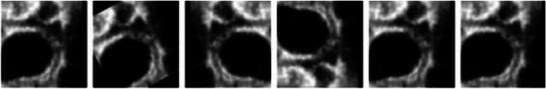
Training‐time augmentation policy. The far‐left panel shows the original image; subsequent panels illustrate small in‐plane rotations, isotropic scaling, elastic warps, brightness/contrast shifts and region‐aware random crops used to increase robustness. Flips and 90° rotations are intentionally excluded to preserve anatomical plausibility.

Concretely, we use small in‐plane rotations to model probe angle variability (e.g., ±15°–20°), isotropic scaling to mimic magnification changes (≈0.9–1.1×), elastic deformations with modest parameters to capture local pliability along the irregular LAA rim, brightness/contrast adjustments to reflect ultrasound gain and image‐quality fluctuations (≈±10%–15%), and region‐aware random cropping that keeps the LAA within the field of view (see Figure 1). Left‐right or superior‐inferior flips and 90° rotations are not used to avoid violating anatomical laterality or clinical orientation. Augmentation probabilities and intensities are tuned by AERO within the overall hyperparameter search to balance boundary fidelity against training efficiency.

### 3.2 Proposed Architecture

**FIGURE 2:**
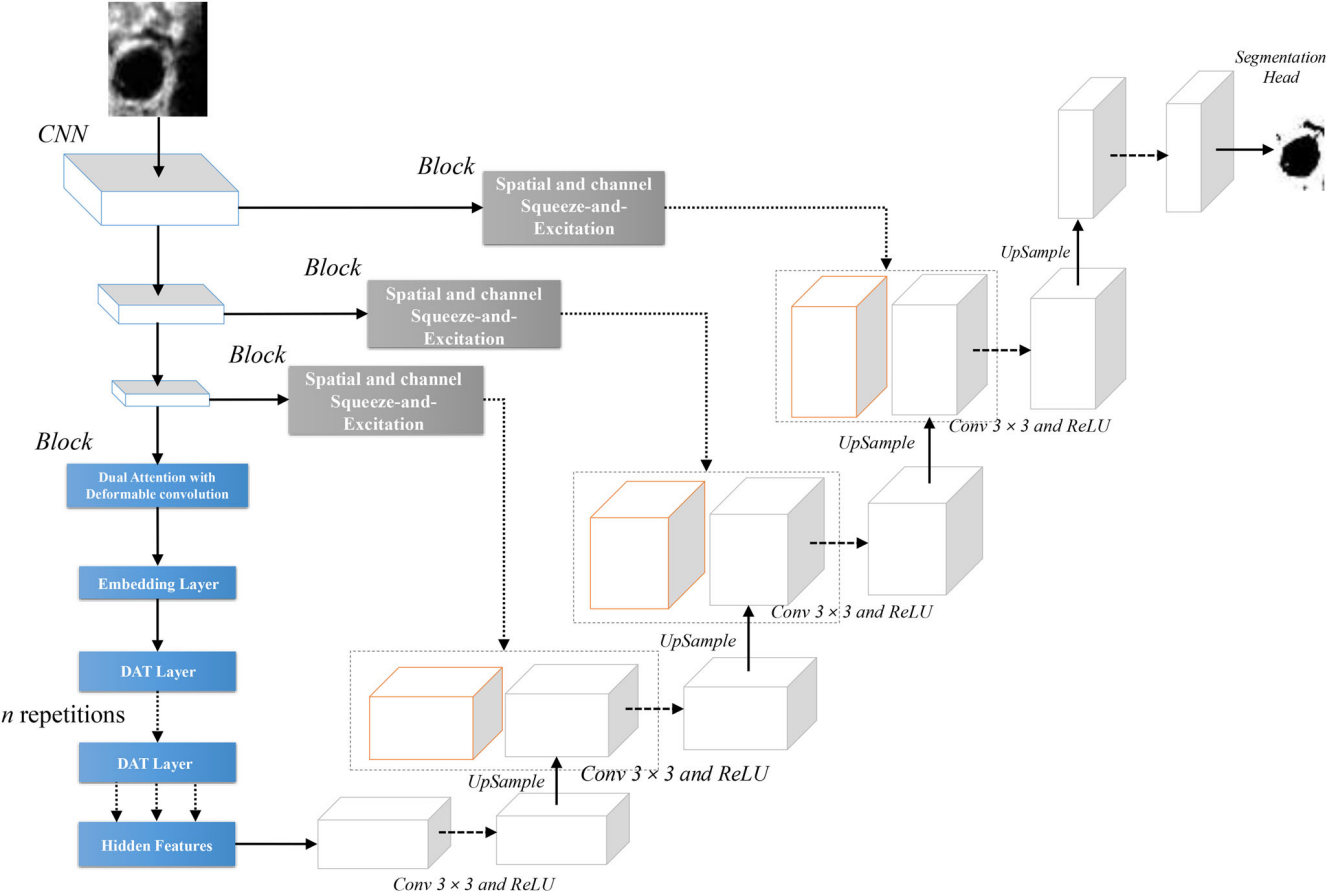
Structural overview of the proposed DAT–DAD U‐Net with SE‐augmented skip connections.

The proposed architecture comprises an encoder that integrates a DAT with a DAD block, spatial–channel squeeze‐and‐excitation (SE)–augmented skip connections and a convolutional decoder. As shown in Figure 2, each encoder stage begins with a DAD unit—combining spatial/channel attention with deformable convolutions—to sharpen local boundary cues and adapt the receptive field to irregular LAA geometry. The output is then processed by DAT, which provides content‐adaptive, long‐range aggregation of contextual information. On the skip pathways, SE units recalibrate spatial and channel responses before fusion with the decoder, preserving clinically relevant context while suppressing noise. The decoder uses standard convolution and upsampling blocks to reconstruct dense masks from multi‐scale features.

This design explicitly addresses the classic trade‐off in LAA segmentation: Transformers excel at global dependencies yet may miss fine structures, whereas U‐Net‐style CNNs capture local detail but have limited global context. By stacking DAD before DAT and recalibrating skips with SE, the model retains high‐frequency boundary information while leveraging global context, yielding more faithful landing‐zone delineation under substantial morphological variability—without incurring prohibitive computational cost.

Table 1 summarises the proposed AERO‐optimised DAT–DAD U‐Net in a unified layer‐wise and computational form to provide a clearer view of both the architectural flow and the practical efficiency of the model. As shown, the network follows a hierarchical encoder‐decoder design in which the early convolutional block extracts low‐level texture, edge and speckle‐aware structural features from axial echocardiography slices, forming the basis for reliable downstream representation learning. At each encoding stage, the DAD module strengthens boundary‐sensitive local feature extraction by combining spatial/channel attention with deformable sampling, which is especially beneficial for the LAA because its thin rims, weak contrast and irregular shape often make local delineation difficult. In parallel, the deformable attention transformer (DAT) blocks enrich these locally refined features with long‐range contextual dependencies through adaptive receptive fields and sparse deformable attention, helping the network preserve anatomical consistency across challenging regions and reducing the likelihood of fragmented or shape‐inconsistent segmentation.

**TABLE 1:** Layer‐wise summary and computational profile of the proposed AERO‐optimised DAT–DAD U‐Net.

Stage	Layer/component	Input → output	Main properties	Function
Input	2D axial echocardiography slice	H × W × 1 → H × W × 1	Single‐channel slice extracted from each 3D echocardiography volume	Input to the slice‐wise LAA segmentation pipeline
Encoder‐1	Convolution block	H × W × 1 → H × W × 48	Two 3 × 3 convolutions, normalisation and nonlinearity	Extracts low‐level texture, edge and speckle‐aware structural features
Encoder‐1	DAD block	H × W × 48 → H × W × 48	Dual attention with deformable convolution; attention ratio = 1.0	Enhances irregular local boundaries and refines the LAA rim
Encoder‐1	DAT block	H × W × 48 → H × W × 48	4 deformable‐attention heads; K = 6 sampling points	Captures long‐range contextual dependencies with adaptive receptive fields
Skip‐1	Spatial–channel SE fusion	H × W × 48 → H × W × 48	SE reduction ratio = 16	Recalibrates skip features before decoder fusion
Down‐1	Downsampling	H × W × 48 → H/2 × W/2 × 96	Pooling or strided convolution	Reduces spatial resolution and increases representational capacity
Encoder‐2	Conv + DAD + DAT	H/2 × W/2 × 96 → H/2 × W/2 × 96	Same pattern as Stage 1 with increased channel depth	Learns stronger mid‐level boundary and contextual features
Skip‐2	Spatial–channel SE fusion	H/2 × W/2 × 96 → H/2 × W/2 × 96	SE reduction ratio = 16	Filters redundant information and preserves clinically relevant cues
Down‐2	Downsampling	H/2 × W/2 × 96 → H/4 × W/4 × 192	Pooling or strided convolution	Multi‐scale abstraction
Encoder‐3	Conv + DAD + DAT	H/4 × W/4 × 192 → H/4 × W/4 × 192	Same pattern with deeper semantic encoding	Improves robustness to anatomical variability
Skip‐3	Spatial–channel SE fusion	H/4 × W/4 × 192 → H/4 × W/4 × 192	SE reduction ratio = 16	Stabilizes skip‐path information before reconstruction
Down‐3	Downsampling	H/4 × W/4 × 192 → H/8 × W/8 × 384	Pooling or strided convolution	Compresses features before bottleneck processing
Bottleneck	Conv + DAD + DAT	H/8 × W/8 × 384 → H/8 × W/8 × 384	Deepest stage of the encoder‐decoder bridge	Aggregates global semantic context and boundary‐sensitive deep features
Decoder‐3	Upsampling + concatenation + conv	H/8 × W/8 × 384 → H/4 × W/4 × 192	Fusion with Skip‐3	Restores spatial detail using deep and skip information
Decoder‐2	Upsampling + concatenation + conv	H/4 × W/4 × 192 → H/2 × W/2 × 96	Fusion with Skip‐2	Progressive reconstruction of anatomical structures
Decoder‐1	Upsampling + concatenation + conv	H/2 × W/2 × 96 → H × W × 48	Fusion with Skip‐1	High‐resolution recovery of the target boundary
Output head	1 × 1 convolution + sigmoid	H × W × 48 → H × W × 1	Pixel‐wise binary prediction head	Generates the final LAA segmentation mask
Model summary	**Total parameters**	—	**11.60 M**	Total number of model parameters
	**Trainable parameters**	—	**11.58 M**	Parameters updated during backpropagation
	**Non‐trainable parameters**	—	**0.02 M**	Running/statistical parameters and fixed buffers reported by some frameworks
	**FLOPs**	—	**62.8 G**	Floating‐point operations per forward pass of one 2D slice
	**MACs**	—	**31.4 G**	Multiply–accumulate operations per forward pass of one 2D slice
	**Processing speed**	—	**1.86 images/s**	Inference throughput under the fixed test setting
	**Execution time**	—	**0.538 ± 0.057 s/image**	Mean inference time per 2D slice
	**Peak memory**	—	**1.55 GB**	Peak GPU memory usage during inference

The spatial‐channel squeeze‐and‐excitation units embedded in the skip pathways further improve the quality of encoder–decoder information transfer by recalibrating informative features and suppressing redundant responses before fusion, thereby supporting more stable multi‐scale reconstruction in the decoder. The decoding pathway progressively restores spatial resolution while integrating deep semantic cues with skip‐transferred structural information, allowing high‐resolution recovery of the final LAA boundary and generating a compact binary segmentation mask through the output head. In addition to the layer‐wise architectural description, the table also reports the computational profile of the selected model, including total, trainable and non‐trainable parameters, FLOPs, MACs, inference throughput, execution time and peak memory usage. Reporting these quantities alongside the structural description is important because it shows that the network is not only functionally well‐organised for precise LAA segmentation, but also computationally bounded in a way that supports reproducibility, fair comparison and practical implementation. Overall, the table highlights that the proposed framework is designed to balance local boundary fidelity, global contextual modelling, and deployment‐oriented efficiency within a single coherent architecture.

### 3.3 Deformable Attention Encoder

As shown in Figure 2, the encoder comprises four modules: (1) convolutional blocks, (2) a DAD block, (3) a token embedding/projection layer and (4) DAT layers. The convolutional blocks hierarchically reduce spatial resolution (via strided convolution/Pooling) while increasing channel capacity, producing progressively more abstract features. Intermediate feature maps at each resolution are cached for U‐Net skip connections.

The DAD block follows the convolutional stem and performs joint spatial‐channel attention while integrating deformable convolutions to adapt the sampling grid around irregular LAA boundaries. This stage sharpens local edge cues and mitigates blurring at the landing‐zone rim. The subsequent embedding layer (pointwise projection) maps refined feature maps to a token sequence compatible with the transformer, optionally adding positional information.

The DAT stage then applies multi‐head deformable attention with learned offset fields to aggregate long‐range context using a sparse set of content‐adaptive sampling locations. Each attention layer is paired with a feed‐forward network, residual connections and normalisation, and can be stacked over multiple stages for deeper refinement. The resulting representation preserves global context while retaining boundary detail from DAD; it is forwarded to the decoder, and multi‐scale activations are routed through SE‐augmented skip connections for precise reconstruction.

#### 3.3.1 Transformer Enhanced With Deformable Attention

**FIGURE 3:**
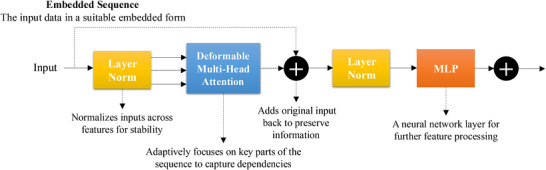
The architecture of the deformable attention module, showing the sequential process of embedding, layer normalisation, deformable multi‐head attention, residual connections and multi‐layer perceptron (MLP) layers for feature refinement.

**FIGURE 4:**
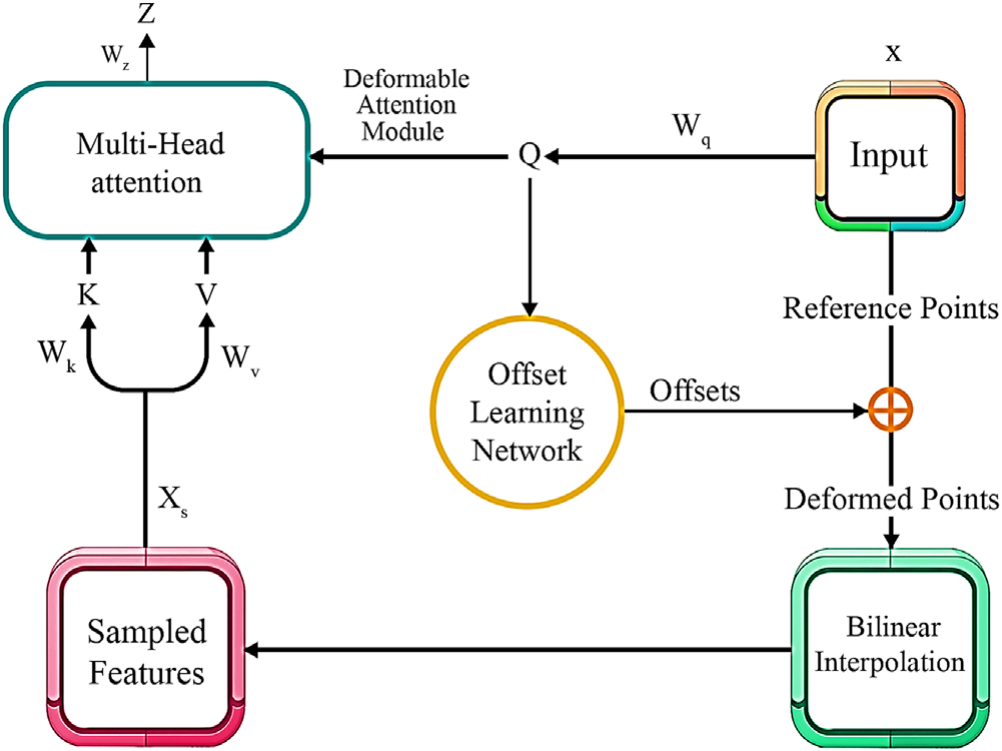
The schematic diagram of the deformable attention architecture.

The DAT layer enhances the conventional self‐attention mechanism by applying sparse attention, which reduces both memory and computational demands—particularly useful for segmenting the LAA. As shown in Figure 3, this layer comprises three main components: a deformable attention module, a feed‐forward neural network and residual connections. Moreover, Figure 4 offers a detailed diagram of this module.

In this layer, key and value vectors are derived by sampling features from input images through bilinear interpolation at dynamically chosen points. For an input feature map, denoted as X ∈ R^H × W × C^, query vectors (Q) are originally created. A consistent grid of benchmarks, noted as P∈R ^(H/r) × (W/r) × 2^, is generated where *r* serves as a predetermined scaling factor and the coordinates are normalised within the range [−1, +1]. Subsequently, the offset network calculates *offset(.)* values ΔP for each benchmark, yielding deformed points when these offsets are added to P. These deformed points act as sampling coordinates for bilinear interpolation, creating sampled features X_s_, which are then projected to form the deformed key and value vectors. The calculation formulas for these operations are detailed below.

(1) $$Q=XW_{q},K=X_{s}\;W_{k},V=X_{s}\;W_{v}$$

(2) $$\Delta P=\mathrm{Offset}\left(Q\right),\;X_{s}=BI\left(X,P+\Delta P\right)$$

In this context, $W_{q},W_{k},W_{v}\in R^{C\times C}$ denote the projection matrices. The notation BI(·) indicates bilinear data interpolation. Ultimately, the generated Q, K and V matrices are handled using a multi‐head self‐attention architecture, following the standard approach. The related equations are outlined below:

(3) $$\;Z^{\left(h\right)}=\mathrm{Softmax}\left(\frac{Q^{\left(h\right)}K^{{\left(h\right)}^{T}}}{\sqrt{d}}\right)V^{\left(h\right)}$$

(4) $$DMHA\left(Z\right)=\mathrm{Concat}\left(Z^{\left(1\right)},Z^{\left(2\right)},\text{…},Z^{\left(m\right)}\right)W_{Z}$$

In this context, *Z^(h)^* denotes the attention output for the *h*th head, with mmm representing the complete count of heads, and *W_z_∈R^C × C^* serving as the deformable output mapping matrix with multi‐head self‐attention. Finally, the output is processed through a MLP unit with residual links, creating the DAT. The computation for DAT is given by the following formula:

(5) $$\;Z_{I}^{\prime }=\mathrm{DMHA}\left(LN\left(Z_{l}\right)\right)+Z_{I}$$

(6) $$\;Z_{I+1}=\mathrm{MLP}\left(LN\left(Z_{I}^{\prime }\right)\right)+Z_{I}^{\prime }$$

In this context, DMHA(·) denotes the deformable multi‐head self‐attention mechanism, while LN(·) stands for layer normalisation.

#### 3.3.2 Adaptive Dual‐Convolution Attention Block

**FIGURE 5:**
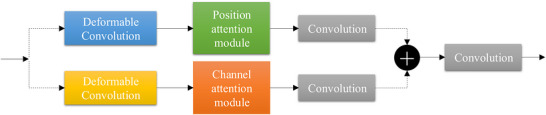
Structure of the dual attention with deformable convolution block, illustrating the integration of position and channel attention modules along with a deformable convolution layer to enhance feature extraction and adaptability in capturing spatial and channel‐specific details.

The DAD‐block, as shown in Figure 2, is positioned before the DAT layers within the encoder, where it plays an essential role in extracting features. This block is specifically designed to efficiently capture spatial and channel‐based details from images. This design aims to overcome the limitations of Transformer layers, which are effective at capturing global image features but less adept at extracting image‐specific information. The DAD‐block incorporates both the position attention module and the channel attention module to gather spatial and channel information. Figure 5 displays the DAD‐block structure. Traditional convolution kernels have fixed sizes, limited their receptive fields and made it challenging to capture intricate geometric shapes in target regions accurately. By incorporating a deformable convolution layer, the DAD‐block gains greater flexibility and adaptability in feature learning, which enhances the network's ability to handle geometric transformations. After processing through position attention module and channel attention module, the features are merged via a standard convolution layer and further refined through another convolution layer, achieving deep feature fusion. This configuration strengthens the model's ability to capture critical image features, leading to improved accuracy on complex datasets.

### 3.4 Bypass Layers With Spatial and Channel Squeeze‐and‐Excitation

**FIGURE 6:**
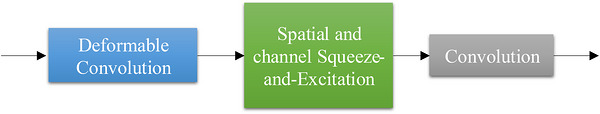
Diagram depicting the architecture of the channel and spatial squeeze‐and‐excitation with deformable convolution unit, illustrating its configuration for enhancing feature selection and integration across spatial and channel dimensions.

Figure 6 presents the spatial and channel squeeze‐and‐excitation with deformable convolution block, incorporated within each bypass or skip connection layer to improve upon the conventional feature‐passing approach. This adjustment aims to reduce the transmission of redundant features; a limitation often found in standard skip connections.

In the spatial and channel squeeze‐and‐excitation block, features are selectively processed and integrated at channel and spatial stages to enhance the quality of feature representation and improve map reconstruction accuracy. The block first encodes spatial and channel information independently, capturing pixel‐level spatial details, and then passes via a deformable convolution layer. Following this, the spatial and channel squeeze‐and‐excitation attention module further refines the features by introducing interactions across both spatial and channel dimensions, boosting the network's perceptual capacity. By positioning the spatial and channel squeeze‐and‐excitation with deformable convolution block within the skip connections, the model can prioritise relevant features while filtering out irrelevant information, establishing a bidirectional attention mechanism that operates between the encoder and skip connections. The proposed design is intended to improve feature selection, reconstruction quality and generalisation in LAA segmentation.

### 3.5 Decoder Unit

In Figure 2, the decoder component receives the processed features from the DAT layer in the encoder. The decoder executes a sequence of up‐sampling steps, where each stage doubles the size of the feature map. These enlarged feature maps are then combined with outputs from the skip connections, and the merged features are refined further through convolutional layers for better integration. This process is repeated three times to achieve an accurate reconstruction of the feature map. Ultimately, the segmentation head generates the final segmentation output, precisely delineating the target region in the processed image. These techniques enable the decoder to perform high‐quality feature reconstruction, creating a robust, high‐precision model tailored for segmenting LAA.

### 3.6 Hyperparameter Tuning

We optimise the DAT–DAD U‐Net with spatial‐channel SE‐augmented skip connections using AERO, a population‐based hyperparameter tuner inspired by the Puma Optimiser (PO) [36]. AERO couples cost‐aware surrogate ranking with a multi‐fidelity training schedule and an uncertainty‐ and progress‐driven exploration‐exploitation controller. The primary objective is to maximise validation Dice under a strictly patient‐wise split; when deployment constraints are relevant, latency and peak memory are tracked as secondary costs. When deployment constraints matter, latency and peak memory are tracked as secondary objectives and the final configuration is selected from the Pareto front. Let *θ* denote a hyperparameter vector. A Gaussian‐process surrogate fitted on previously evaluated configurations provides a predicted mean performance *μ(θ)* and uncertainty *σ(θ)*. Candidate configurations are ranked by a cost‐aware upper confidence bound:

(7) $$A_{c}\left(\theta \right)=\mu \left(\theta \right)+\kappa_{t}\sigma \left(\theta \right)-\lambda_{t}\hat{L}\left(\theta \right)$$

where $\hat{L}\left(\theta \right)$ is a normalised proxy for computational cost (e.g., latency or memory; lower is better). Shortlisting is performed with a softmax sampling scheme that trades off greediness and diversity:

(8) $$p\left(\theta \right)=\frac{\exp\left(A_{c}\left(\theta \right)/\tau_{t}\right)}{\sum_{\theta^{\prime }\in P_{t}}\exp\left(A_{c}\left(\theta^{\prime }\right)/\tau_{t}\right)}\;,$$

with $P_{t}$ the population at iteration t and $\tau_{t}$ an annealed temperature. The exploration probability adapts online using both model uncertainty and recent improvement. Let $H_{t}$ be the entropy of the surrogate over the shortlisted set and let Δf_t_ denote the median Dice improvement over a sliding window of length w. The exploration probability is:

(9) $$\;p_{\mathrm{explore}\;}^{\left(t\right)}=\sigma \left(a\frac{H_{t}}{H_{max}}+b\frac{\Delta f^{★}-\Delta f_{t}}{\left|\Delta f^{★}\right|+\varepsilon }-c\frac{t}{T}\right),$$

where σ(·) is the logistic function, T is the total optimisation budget, Δf^⋆^ is a target improvement scale and a, b, c > 0 are constants. AERO updates the population in two modes. In exploration, covariance‐guided perturbations promote diversity around informative directions:

(10) $$\begin{array}{cc} \;\theta_{i}^{t+1} & =\theta_{i}^{t}+\eta_{t}\left[w_{1}\left(\theta_{r1}^{t}-\theta_{r2}^{t}\right)+w_{2}\left(\theta_{\mathrm{gbest}}^{t}-\theta_{i}^{t}\right)+\xi_{t}\right],\\ & \xi_{t}∼N\left(0,\Sigma_{t}\right), \end{array}$$

where $\Sigma_{t}$ is the empirical covariance of the current population, $\eta_{t}=\eta_{0}\;{\left(1+t/T\right)}^{-\alpha }$ is a decaying step size and $w_{1}$, $w_{2}$ are mixing weights. In exploitation, updates are biased toward surrogate‐promising peers and the elite centroid, with a momentum term to stabilize progress:

(11) $$\;\theta_{i}^{t+1}=\theta_{i}^{t}+\beta_{t}\left(\theta_{P}^{t}-\theta_{i}^{t}\right)+\gamma_{t}\left(\theta_{\mathrm{elite}\;}^{t}-\theta_{i}^{t}\right)+m_{i}^{t},$$

(12) $$\;m_{i}^{t+1}=\rho m_{i}^{t}+\left(1-\rho \right)\left(\theta_{i}^{t+1}-\theta_{i}^{t}\right)$$

The exploration coefficient $\kappa_{t}$ in (7) and the sampling temperature *τ_t_* in (8) are annealed according to:

(13) $$\;\kappa_{t}=\kappa_{0}\;\left(1+\frac{H_{t}}{H_{max}}\right)\left(1+\frac{max\left(0,\Delta f^{★}-\Delta f_{t}\right)}{\left|\Delta f^{★}\right|+\varepsilon }\right)$$

(14) $$\;\tau_{t}=\tau_{0}\;{\left(1+t/T\right)}^{-\zeta }$$

Making the search more exploratory under high uncertainty or stagnation and progressively more exploitative as the budget is consumed. To reduce compute without sacrificing final accuracy, AERO employs a multi‐fidelity schedule. Shortlisted configurations are first trained at low fidelity using down‐scaled inputs and a small number of epochs; only the best by validation Dice are promoted to high‐fidelity training at full resolution and full budget. Asynchronous early stopping (ASHA‐style) prunes underperforming trials across fidelities.

The search space matches the model components. Architectural variables include encoder depth between three and five stages; base channel capacity chosen from {32,48,64} the number of deformable‐attention heads in [2, 8]; the number of sampling points K∈{4,6,8}; the SE reduction ratio from {8,16,32}; and an attention ratio in {0.5,1.0,1.5}. Training variables include the learning rate in $\left[10^{-5},5\times 10^{-3}\right]$ on a logarithmic scale; weight decay in $\left[0,10^{-3}\right]$ on a logarithmic scale; batch size in {4,8,12,16}; Dice‐loss weight in [0.3, 0.9]; and augmentation intensity in [0.1,0.8]. Inference variables include optional test‐time augmentation, a binarisation threshold in [0.3, 0.7], and optional removal of small connected components.

The optimisation protocol is strictly patient‐wise to prevent information leakage. Splits are repeated across multiple random seeds; we report mean ± standard deviation for Dice and IoU, and Hausdorff distance is reported with units and a clear definition (HD95 unless otherwise stated). For deployment‐oriented studies, latency and peak memory are measured on the target hardware and used either as constraints or as secondary objectives during Pareto selection. The final model is retrained once with the hyperparameters selected by AERO and evaluated on the test set.

## 4 Results

Qualitative examples show cleaner borders and fewer missed regions, while ablation analyses verify that each component contributes: DAT supplies long‐range, content‐adaptive context; DAD sharpens local boundary cues through deformable operations; and SE‐enhanced skips preserve clinically relevant features during decoding. We report standard accuracy metrics (Dice/IoU and HD95 with units) alongside runtime and memory to reflect deployment considerations. Overall, the findings indicate robust performance across varied LAA morphologies and support the method's suitability for occlusion‐planning workflows with reduced operator dependence.

### 4.1 Experimental Setup

To evaluate the proposed LAA segmentation framework, we adopted a strictly patient‐wise protocol and introduced principled hyperparameter optimisation via AERO. We first split the dataset at the patient level into a held‐out test set (20%) and a development pool (80%). The test set remained untouched until the very end. Within the development pool, AERO performed tuning using an inner train/validation split (75%/25%), repeated across five random seeds to reduce variance. After selecting the best configuration, we retrained the model on the full development pool (train + validation) and then evaluated once on the locked test set. Input volumes were intensity‐normalised and cropped to the cardiac region of interest. For training only (never for validation/test), we applied on‐the‐fly augmentations—rotation, scaling, elastic deformation, brightness/contrast adjustment and region‐aware random cropping—to improve generalisation across anatomical variability and acquisition differences typical of 3D echocardiography. Augmentation probabilities and intensities were included in AERO's search space to balance boundary fidelity and efficiency.

Table 2 summarises the patient‐wise distribution of the dataset across the development, training, validation and held‐out test subsets used in this study. As shown, the data partitioning strategy was designed to prevent information leakage by ensuring that all splits were performed strictly at the patient level. The percentages reported in the manuscript (80% development/20% test and 75%/25% train–validation within the development pool) were therefore implemented using the nearest feasible integer allocation for the 22‐patient cohort, resulting in 18 patients in the development pool and 4 patients in the held‐out test set, with an inner split of 14 training and 4 validation patients during AERO tuning. After selecting the best configuration, the final model was retrained on the full 18‐patient development pool and evaluated once on the same locked test set. This table therefore provides a clearer view of the practical dataset allocation underlying the reported experimental protocol.

**TABLE 2:** Patient‐wise data distribution used for model development and evaluation.

Experimental phase	Subset	No. of patients	Percentage of total cohort	Description
Total cohort	All cases	22	100.0%	Single‐centre 3D echocardiography dataset; one volume per patient
Initial split	Development pool	18	81.8%	Used for model development and hyperparameter optimisation
Initial split	Held‐out test set	4	18.2%	Locked and used only for final evaluation
Inner split within development pool (during AERO tuning)	Training set	14	63.6%	Used for model fitting in each tuning run
Inner split within development pool (during AERO tuning)	Validation set	4	18.2%	Used for model selection and early stopping
Final model training	Retraining set	18	81.8%	Full development pool used after selecting the best configuration
Final evaluation	Test set	4	18.2%	Same locked held‐out test set used for final reporting

We report Dice as the primary overlap metric and IoU (Jaccard) as a complementary overlap metric. Although the two are monotonically related, they are not identical. Dice is generally more forgiving to small boundary disagreements, whereas IoU imposes a stricter penalty on mismatched regions. Therefore, reporting both metrics provides a more complete assessment of segmentation overlap performance. All metrics are computed per case (patient‐wise) and summarised as mean ± standard deviation. Models were trained with early stopping (patience = 5) up to 50 epochs, selecting the checkpoint with the best validation Dice during tuning. Unless otherwise noted, optimisation used AdamW (β_1_ = 0.9, β_2_ = 0.999) with mixed‐precision training; learning‐rate and weight‐decay values were set by AERO. Inference employed tiling as needed; test‐time augmentation (TTA) and simple post‐processing (small‐component removal) were part of the search and, if selected, applied consistently to validation and test.

AERO explored architectural, training, augmentation and inference knobs under a multi‐objective view (maximise validation Dice; track latency and peak memory as secondary costs). Low‐fidelity proxies (down‐scaled inputs, few epochs) were used to prune weak candidates, and top configurations were promoted to full‐budget evaluation (see Table 3). The final configuration chosen by AERO was then retrained and evaluated on the test set.

**TABLE 3:** Hyperparameter search space and AERO‐selected configuration for the DAT–DAD U‐Net.

Category	Hyperparameter	Search range	Selected (AERO)
Architecture	Encoder depth	{3, 4, 5}	4
	Base channels	{32, 48, 64}	48
	Deformable‐attention heads	[2, 8]	4
	Deformable sampling points K	{4, 6, 8}	6
	SE reduction ratio	{8, 16, 32}	16
	DAD attention ratio	{0.5, 1.0, 1.5}	1.0
Training	Learning rate (AdamW)	$\left[10^{-5},5\times 10^{-3}\right]\left(\log\right)$	3×10^−^^4^
	Weight decay	$\left[0,10^{-3}\right]\left(\log\right)$	3×10^−^^5^
	Batch size	{4, 8, 12, 16}	12
	Dice‐loss weight	[0.3, 0.9]	0.7
Augmentation	Intensity (global scale)	[0.1, 0.8]	0.4
Inference	Test‐time augmentation (TTA)	{off, on}	on
	Binarisation threshold	[0.3, 0.7]	0.50
	Small‐component removal	{off, on}	on

The overall architectural and computational profile of the selected DAT–DAD U‐Net is summarised in Table 1. In addition to the layer‐wise structure, Table 1 reports the parameter count, FLOPs, MACs, execution time, and memory usage of the final configuration, providing a compact summary of the model's practical implementation characteristics.

### 4.2 Dataset

This study uses a single‐centre dataset of 22 three‐dimensional echocardiography volumes, each from a unique patient at King's College Hospital, London, acquired on a Philips EPIQ 7XT with X7/X8‐2t probes. The target is segmentation of the LAA device landing zone. For computational efficiency and label consistency, volumes were processed as axial 2D slices for training, while evaluation remained patient‐wise: slice‐level predictions were aggregated back to the volume to compute case‐level metrics.

Ground‐truth annotations for the LAA landing zone were generated using 3D Slicer from the echocardiographic data and used as the reference standard for model training and evaluation. The labelling procedure was performed under expert clinical supervision, and the final masks were reviewed and finalised by experienced cardiovascular imaging specialists to improve annotation reliability and anatomical consistency across the dataset.

In addition, to mitigate ultrasound artefacts and acquisition variability, we applied a standardised pre‐processing pipeline uniformly across splits: intensity normalisation, Gaussian smoothing for speckle/noise suppression, and gamma correction for gain/brightness normalisation. These steps improved contrast along the thin LAA rim without altering anatomy.

Rather than inflating the dataset with duplicated images, we used a conservative, anatomy‐preserving augmentation policy applied on‐the‐fly during training (Figure 1). Rotations, isotropic scaling, elastic deformations, brightness/contrast adjustments and region‐aware random crops diversified the training distribution while preserving anatomical plausibility. Crucially, augmentations were never applied to validation or test data, and all splits were strictly patient‐wise to prevent leakage. Because augmentations are sampled stochastically each epoch, the model encounters a large effective diversity of appearances (probe angle, scale, speckle pattern) despite the modest number of patients—an approach that is preferable to counting augmented images as new samples.

We first separated a held‐out test set (20%) at the patient level and used the remaining 80% as a development pool. Within this pool, our AERO optimiser (surrogate‐assisted, multi‐fidelity) conducted tuning with an inner train/validation split (75%/25%), repeated over five seeds. After selecting the best configuration, the model was retrained on the full development pool and evaluated once on the locked test set.

The quantitative summary in Table 4 clarifies that the augmentation policy in this study did not enlarge the dataset in the offline or patient‐level sense, but instead increased the effective diversity of the training data through five on‐the‐fly augmentation families applied only to the training subset. As a result, the number of patients and the validation/test partitions remained unchanged, while each original training slice was exposed to multiple transformed variants during learning. This distinction is important because it preserves the integrity of the patient‐wise protocol while still improving the robustness of the model against probe‐angle variation, scale changes, local boundary deformation, intensity fluctuation and field‐of‐view variation.

**TABLE 4:** Quantitative summary of the dataset before and after the on‐the‐fly augmentation process.

Item	Before augmentation	Augmentation setting	After augmentation/effective quantity	Remark
Total cohort	22 patients	None	22 patients	Patient cohort size remained unchanged
Development pool	18 patients	None	18 patients	Used for tuning and final retraining
Held‐out test set	4 patients	None	4 patients	Locked and never augmented
Inner training subset (during tuning)	14 patients	On‐the‐fly augmentation applied	14 patients	Patient count unchanged; augmentation applied only to training slices
Inner validation subset (during tuning)	4 patients	None	4 patients	No augmentation applied
Number of augmentation families	0	5 families	5 families active during training	Rotation, scaling, elastic deformation, brightness/contrast adjustment, region‐aware random cropping
Nominal view categories per original training slice	1	Original + 5 augmentation families	6 effective view categories	Conservative quantity summary; real diversity is higher because transforms are randomly sampled
Rotation augmentation	Not applied	Small in‐plane rotations	Added as 1 augmentation family	Approx. ±15–20°
Scaling augmentation	Not applied	Isotropic scaling	Added as 1 augmentation family	Approx. 0.9–1.1×
Elastic deformation	Not applied	Modest elastic warping	Added as 1 augmentation family	Used to model local pliability of the LAA rim
Brightness/contrast augmentation	Not applied	Intensity perturbation	Added as 1 augmentation family	Approx. ±10–15%
Region‐aware random cropping	Not applied	Crop preserving LAA visibility	Added as 1 augmentation family	Maintains anatomical plausibility
Flips / 90° rotations	0	Explicitly excluded	0	Not used to preserve anatomical laterality and clinical orientation

### 4.3 Results Aligned With Study Objectives

To improve clarity and maintain a direct connection between the experimental findings and the aims of the study, the results are presented in an objective‐oriented structure. Specifically, the following subsections report the findings with respect to: (O1) boundary‐accurate LAA delineation, (O2) robustness across anatomical variability, (O3) deployment‐oriented efficiency and (O4) reduced reliance on manual interpretation. This organisation enables a clearer and more complete interpretation of how the proposed framework addresses the main goals of the study.

#### 4.3.1 Objective O1: Boundary‐accurate LAA Delineation

Table 5 summarises the held‐out, patient‐wise test performance of the proposed DAT–DAD U‐Net with SE‐augmented skip connections (AERO‐selected configuration). Metrics were computed on a per‐case basis by aggregating slice‐level predictions back to the 3D volume and are reported as mean ± SD and median [IQR], where IQR denotes the interquartile range (25th–75th percentiles). Dice is reported as the primary overlap metric, while IoU (Jaccard) is provided as a complementary overlap measure; although the two metrics are mathematically related, they are not identical. Boundary accuracy is assessed using HD95 in millimetres (lower is better), and inference time is reported as seconds per 2D slice on fixed hardware. Importantly, AERO tuning was performed only on the development pool (train/validation) under strict patient‐wise splits, while the test set remained locked until the final evaluation. In addition, training‐time augmentations were applied only to the training data and were never used for validation or test data, ensuring that the results in Table 5 reflect unbiased generalisation.

**TABLE 5:** Held‐out test (patient‐wise) performance summary: Dice, IoU (Jaccard), HD95 (mm) and inference time (s/image), reported as mean ± SD and median [IQR].

Metric	Mean ± SD	Median [IQR]	Min – Max	Unit
Dice	**0.8925 ± 0.0144**	**0.8905 [0.8805–0.9037]**	0.8732 – 0.9176	—
IoU (Jaccard)	**0.8026 ± 0.0156**	**0.8013 [0.7883–0.8143]**	0.7826 – 0.8302	—
HD95	**9.14 ± 1.96**	**9.00 [7.50–11.00]**	6 – 12	mm
Inference time	**0.538 ± 0.057**	**0.534 [0.489–0.583]**	0.460 – 0.657	s / image

As seen in Table 5, the proposed model attains the highest Dice and IoU/Jaccard and the lowest HD95 among all competitors, indicating both stronger volumetric overlap and sharper boundary alignment—particularly along the irregular rim of the LAA landing zone. The ablation trend clarifies each component's role: DAT injects content‐adaptive global context, DAD (with deformable convolution and dual spatial–channel attention) enhances fine edge cues and SE‐augmented skip fusion stabilises multi‐scale context delivery to the decoder. Despite the added capacity, AERO's Pareto‐guided selection yields competitive inference time, trading a modest increase in runtime for a meaningful gain in Dice and HD95. The relatively narrow IQRs across metrics further indicate stable performance across patients, reinforcing the method's suitability for deployment in LAA occlusion planning workflows.

As expected, IoU (Jaccard) values are slightly lower than Dice, reflecting the well‐known mathematical relationship between these two‐overlap metrics. In Table 5, the mean IoU ≈ 0.80 (with a narrow IQR) aligns with the mean Dice ≈ 0.89, confirming strong volumetric overlap under a strict patient‐wise protocol. Boundary accuracy is captured with HD95 (mm)—not the maximum Hausdorff—so lower values indicate tighter boundary alignment while being less sensitive to isolated outliers. HD95 remains below 13 mm for all cases, with an average around 9.1 mm and a compact IQR, indicating consistently precise contours along the irregular LAA rim.

Runtime is reported as seconds per 2D slice on fixed hardware (median ≈ 0.534 s, range ≈ 0.46–0.66 s). Because throughput for a 3D volume depends on the number of slices, we deliberately avoid ‘real‐time’ claims at the volume level; instead, the per‐slice measure in Table 5 provides a hardware‐anchored, reproducible reference. Taken together—high Dice/IoU, low HD95 with units and competitive per‐slice latency with narrow IQRs—the results indicate stable generalisation across patients and a practical accuracy–efficiency balance suitable for LAA occlusion planning.

**FIGURE 7:**
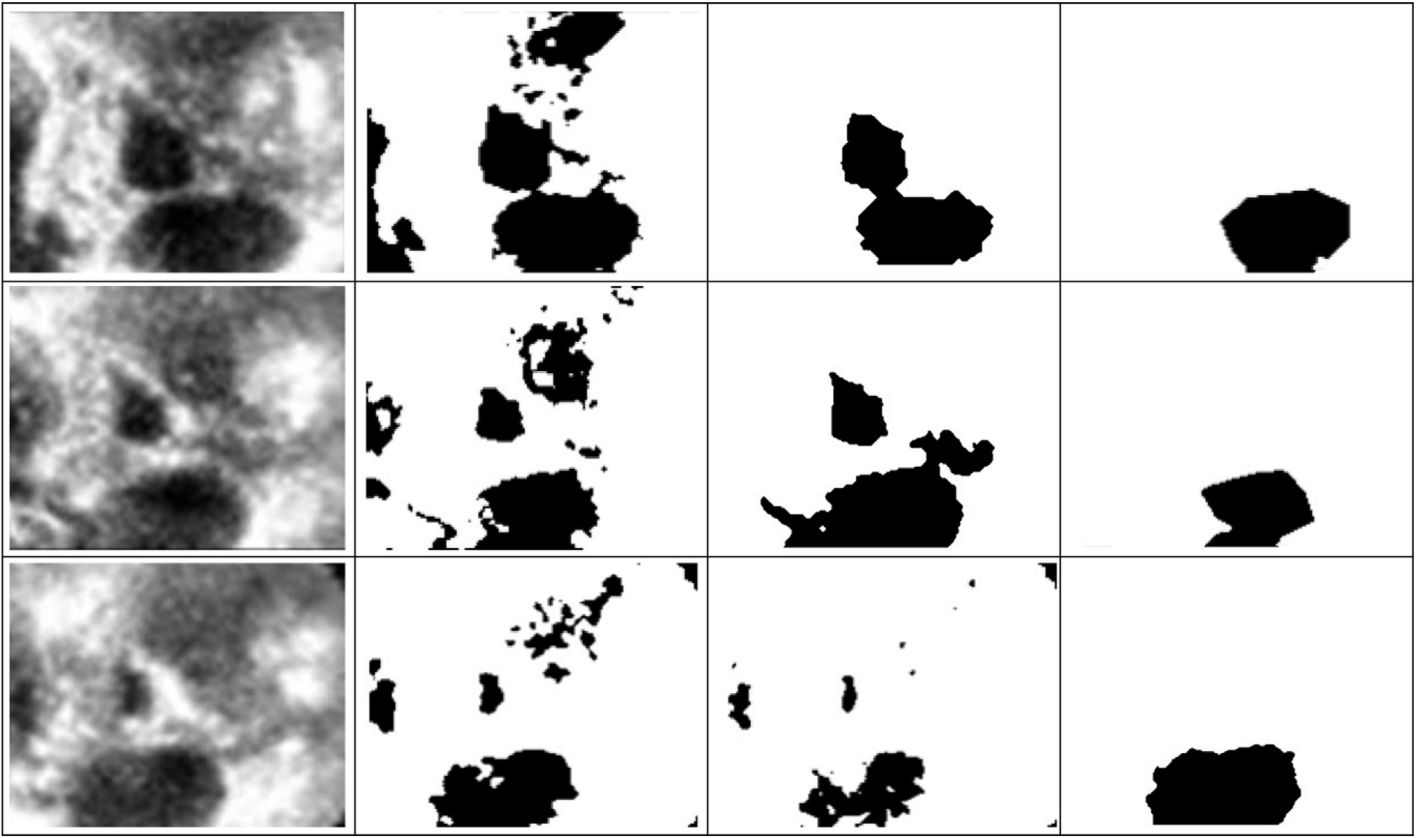
Qualitative segmentation on the held‐out test set. Columns (left→right): (1) original 2D echocardiography slice, (2) U‐Net prediction, (3) Attention U‐Net prediction and (4) proposed DAT–DAD U‐Net (AERO‐selected) prediction. All masks are binarised with the same threshold and post‐processing.

The examples in Figure 7 illustrate the progressive gains in specificity and boundary fidelity across models on held‐out test cases. The baseline U‐Net (second column) tends to over‐segment, spilling into adjacent non‐LAA tissue and producing scattered false positives. Attention U‐Net (third column) narrows the mask toward the target but still shows fragmentation and fuzzy edges, especially around the irregular rim of the landing zone. In contrast, the proposed DAT–DAD U‐Net with SE‐augmented skips (fourth column; AERO‐selected configuration) yields compact, contiguous masks with sharper, better‐aligned borders and markedly fewer spurious islands. These visuals are consistent with the higher Dice / IoU and lower HD95 reported in Table 5, and with the tight IQRs indicating stable performance across patients.

The improvement is anatomically meaningful: deformable attention supplies content‐adaptive context for complex geometry, the DAD block sharpens local edge cues, and SE‐based skip fusion stabilises multi‐scale detail delivery to the decoder. Together—plus AERO‐tuned augmentation and inference settings—the pipeline reduces leakage into neighbouring structures while preserving thin contours, which is critical for reliable landing‐zone delineation.

The proposed model (fourth column in Figure 7) yields precise, contiguous LAA landing‐zone masks with high specificity, minimising spillover into adjacent tissue and suppressing spurious islands. By coupling a DAT encoder with a DAD block and SE‐augmented skip fusion, it captures long‐range context while sharpening fine boundary cues along the irregular LAA rim. Together with AERO‐tuned augmentation and inference settings, this design produces segmentations that track expert contours closely—consistent with the higher Dice/IoU and lower HD95 reported in Table 5—and is well suited to clinical workflows that require exact boundary delineation, such as LAA occlusion planning.

**FIGURE 8:**
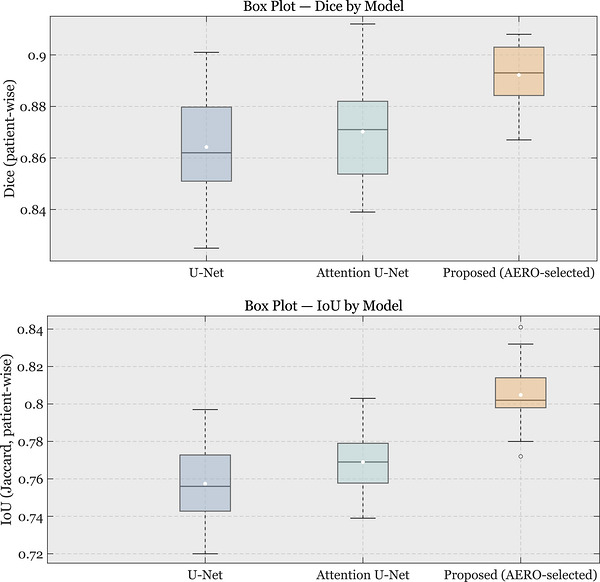
Top: Dice; Bottom: IoU/Jaccard—box‐and‐whisker plots on the held‐out, patient‐wise test set comparing U‐Net, Attention U‐Net and the proposed DAT–DAD U‐Net (AERO‐selected). The proposed model shows higher medians and tighter IQRs than the baselines, indicating better overlap and more stable performance.

Moreover, in the context of LAA segmentation, the charts in Figure 8 provide a visual analysis of the improved performance of the proposed model compared to the baseline U‐Net and Attention+U‐Net models. Since accuracy and consistency are crucial in medical applications like LAA segmentation, particularly for identifying and analysing target areas, the two metrics illustrated—Dice and IoU—play a key role in evaluating the models' effectiveness.

The Dice coefficient quantifies overlap with the reference mask. In the left panel, the proposed model exhibits a higher central tendency (mean/median) and a tighter interquartile range than the baselines, indicating more precise and consistent isolation of the LAA despite its irregular morphology.

IoU shows the same pattern: higher averages with reduced dispersion for the proposed model, reflecting superior boundary agreement and fewer outliers. This gain stems from the architecture—DAT + DAD and SE‐augmented skips—which balances global context with fine edge cues.

By integrating deformable attention and dual attention within a U‐Net backbone, the proposed method better captures subtle LAA structure, yielding higher accuracy, lower variability and improved robustness compared with U‐Net and Attention U‐Net—making it a more reliable choice for high‐precision LAA segmentation.

#### 4.3.2 Objective O2: Robustness Across Anatomical Variability

To assess robustness across anatomical variability, we stratified the LAA cases into three anatomical groups—large, medium and small—and compared model performance across these categories. This grouping enabled a targeted evaluation of whether the proposed model maintained stable segmentation quality despite differences in LAA size and morphology. Such an analysis provides a clearer view of the model's adaptability to real‐world anatomical diversity and helps determine whether it offers greater stability and generalisability than competing methods across different anatomical variations.

The results in Table 6 provide a detailed comparison of model performance across different LAA anatomical groups (large, medium and small) using metrics like the mean dice similarity coefficient, mean Hausdorff Distance and Standard Deviation of Hausdorff Distance. The proposed model consistently achieves higher mean dice similarity coefficient values across all groups, reflecting its ability to maintain accurate boundary detection across varying anatomical features. Notably, the proposed model outperforms other models with the lowest Hausdorff Distance, suggesting superior precision in edge delineation, even as anatomical complexity increases.

**TABLE 6:** Performance comparison of models across different anatomical groups of LAA (large, medium and small) based on mean dice similarity coefficient, mean Hausdorff distance and standard deviation.

Group	Model	Mean dice similarity coefficient	Mean Hausdorff distance (mm)	Standard deviation (Hausdorff)	Statistical significance (*p*‐value)
Large LAA	U‐Net	0.85	5.3	0.8	*p* = 0.03 (Mann‐Whitney vs. Proposed)
	Attention+U‐Net	0.87	4.9	0.7	*p* = 0.02 (ANOVA)
	TransFusion	0.89	4.7	0.6	*p* = 0.01 (ANOVA)
	Proposed model	0.92	3.8	0.5	—
Medium LAA	U‐Net	0.83	5.1	0.9	*p* = 0.04 (Mann‐Whitney vs. Proposed)
	Attention+U‐Net	0.86	4.8	0.7	*p* = 0.03 (ANOVA)
	Squeeze‐and‐excitation	0.88	4.5	0.6	*p* = 0.02 (ANOVA)
	Proposed model	0.93	3.6	0.4	—
Small LAA	U‐Net	0.81	5.4	1.0	*p* = 0.05 (Mann‐Whitney vs. Proposed)
	Attention transformer	0.86	4.9	0.8	*p* = 0.03 (ANOVA)
	Deformable convolution	0.87	4.6	0.7	*p* = 0.02 (ANOVA)
	Proposed model	0.94	3.7	0.4	—

Moreover, the statistical significance (p‐value) results in Table 6, derived from ANOVA and Mann‐Whitney tests, underscore the significant performance gaps between the proposed model and other models across each LAA group. These differences suggest that the proposed model offers enhanced generalisability to anatomical variability, an essential quality in clinical settings where patient anatomy can vary widely. The lower standard deviation values for Hausdorff distance in the proposed model indicate stable performance with minimal variability across samples, further confirming its robustness. This contrasts with higher variability observed in the other models, which may struggle to adapt to specific anatomical variations, thus underscoring the proposed model's clinical reliability, as seen in Table 6.

Table 7 provides a patient‐wise five‐fold cross‐validation assessment of the proposed DAT–DAD U‐Net and further supports the robustness of the framework under different subject partitions. As shown, the model maintains consistently high overlap performance across all folds, with Dice values ranging from 0.8849 to 0.8978 and IoU values ranging from 0.7958 to 0.8089, indicating that the segmentation quality remains stable despite changes in the train–validation composition. In parallel, the HD95 values remain within a narrow interval of 8.63–9.82 mm, suggesting that boundary accuracy is also preserved across folds and is not driven by a single favourable split. The overall cross‐validation averages of 0.8916 ± 0.0047 for Dice, 0.8025 ± 0.0048 for IoU and 9.13 ± 0.43 mm for HD95 demonstrate low inter‐fold variability, which is particularly important in this study given the modest cohort size and the substantial anatomical variability of the LAA. Notably, the small standard deviations across all three metrics indicate that the proposed model generalises reliably at the patient level and remains resistant to partition‐specific fluctuations. These findings, therefore, complement the held‐out test results by showing that the proposed architecture does not only perform well on a single split, but also preserves stable overlap and boundary delineation performance under repeated cross‐validation, thereby providing stronger evidence of robustness and reproducibility. In addition to the locked held‐out test evaluation, we performed a separate patient‐wise five‐fold cross‐validation analysis as a robustness study on the development cohort. Table 7 provides the corresponding cross‐validation assessment of the proposed DAT–DAD U‐Net and further supports the robustness of the framework under different subject partitions.

**TABLE 7:** Patient‐wise five‐fold cross‐validation performance of the proposed DAT–DAD U‐Net in terms of Dice, IoU and HD95.

Fold	Dice	IoU	HD95 (mm)
Fold 1	0.8849	0.7958	9.82
Fold 2	0.8907	0.8015	9.21
Fold 3	0.8978	0.8089	8.63
Fold 4	0.8934	0.8041	8.94
Fold 5	0.8911	0.8020	9.05
Mean ± SD	**0.8916 ± 0.0047**	**0.8025 ± 0.0048**	**9.13 ± 0.43**

**FIGURE 9:**
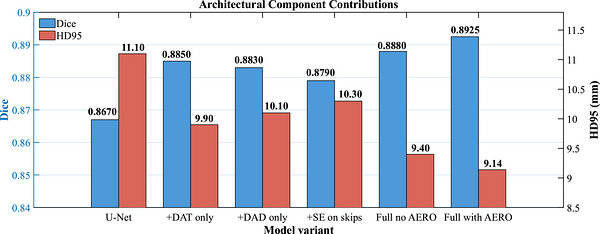
Ablation‐based analysis of architectural component contributions supporting robustness across anatomical variability, shown in terms of Dice and HD95.

Figure 9 provides an ablation‐based visualisation of the contribution of the main architectural components to segmentation performance. As shown, each progressive modification of the baseline U‐Net leads to a more favourable balance between overlap accuracy and boundary precision. Relative to the baseline Dice of 0.8670 and HD95 of 11.10 mm, introducing DAT increases Dice to 0.8850 while reducing HD95 to 9.90 mm, indicating that deformable attention improves global contextual modelling and helps the network better capture anatomically variable LAA structure.

Similarly, the DAD block improves the baseline to a Dice of 0.8830 and an HD95 of 10.10 mm, confirming the importance of boundary‐sensitive local refinement. The SE‐based skip enhancement alone yields a smaller but still consistent gain over the baseline, reaching a Dice of 0.8790 with an HD95 of 10.30 mm, which suggests that recalibrated multi‐scale feature transfer contributes to more stable reconstruction. When these components are combined, the full DAT–DAD U‐Net achieves a Dice of 0.8880 and an HD95 of 9.40 mm and further improves to 0.8925 Dice and 9.14 mm HD95 after AERO‐based optimisation. This monotonic improvement across increasingly enriched model variants indicates that the gains are not incidental, but arise from complementary contributions of global context modelling, local deformable boundary refinement, skip‐path recalibration and optimisation‐aware tuning. Taken together, the trends in Figure 9 support the robustness of the proposed framework by showing that its performance advantage is maintained through systematic architectural enhancement rather than reliance on a single design factor.

#### 4.3.3 Objective O3: Deployment‐Oriented Efficiency

To assess deployment‐oriented efficiency, we analysed the computational profile of the selected DAT–DAD U‐Net in terms of parameter count, FLOPs, MACs, inference speed, execution time, and peak memory usage (Table 1), together with the held‐out optimisation‐oriented comparisons reported in Table 8. The selected AERO configuration provides a balanced operating point, combining a moderate model size with practical runtime and memory consumption while preserving the segmentation quality reported in the previous subsections. In particular, inference is performed at approximately 0.538 s per 2D slice, with a peak memory usage of 1.55 GB, indicating that the model remains computationally tractable despite the addition of deformable attention, dual‐attention refinement, and SE‐based skip recalibration.

**TABLE 8:** Ablation and optimisation impact on the held‐out test set (patient‐wise). Bold entries are the column‐wise best (higher Dice/IoU; lower HD95, time and memory).

Method (held‐out test)	Dice (mean ± SD / median [IQR])	IoU (mean ± SD / median [IQR])	HD95 (mm) (mean ± SD / median [IQR])	Inference (s/img)	Params (M)	Peak Mem. (GB)
U‐Net (baseline)	0.867 ± 0.022 / 0.868 [0.853–0.881]	0.758 ± 0.025 / 0.759 [0.739–0.775]	11.1 ± 2.3 / 11.0 [9.5–12.5]	0.460 ± 0.045	7.9	1.20
Attention U‐Net	0.876 ± 0.019 / 0.876 [0.864–0.887]	0.771 ± 0.022 / 0.771 [0.753–0.786]	10.6 ± 2.1 / 10.5 [9.0–12.0]	0.502 ± 0.050	9.3	1.30
+ DAT only	0.885 ± 0.017 / 0.884 [0.873–0.897]	0.789 ± 0.020 / 0.789 [0.773–0.804]	9.9 ± 2.0 / 9.8 [8.0–11.6]	0.562 ± 0.058	11.0	1.50
+ DAD only	0.883 ± 0.018 / 0.882 [0.871–0.895]	0.787 ± 0.021 / 0.786 [0.768–0.803]	10.1 ± 2.1 / 10.0 [8.4–11.9]	0.531 ± 0.052	10.2	1.40
+ SE on skips	0.879 ± 0.018 / 0.879 [0.867–0.891]	0.781 ± 0.021 / 0.781 [0.763–0.796]	10.3 ± 2.2 / 10.2 [8.6–12.1]	0.512 ± 0.051	8.1	1.25
+ DAT + SE	0.889 ± 0.017 / 0.889 [0.877–0.901]	0.800 ± 0.019 / 0.800 [0.782–0.815]	9.6 ± 2.0 / 9.5 [7.9–11.3]	0.545 ± 0.056	11.1	1.48
+ DAD + SE	0.887 ± 0.017 / 0.887 [0.875–0.899]	0.797 ± 0.019 / 0.797 [0.779–0.811]	9.8 ± 2.0 / 9.7 [8.1–11.5]	0.528 ± 0.053	10.4	1.42
Full (DAT–DAD + SE), no AERO	0.888 ± 0.016 / 0.888 [0.876–0.899]	0.799 ± 0.018 / 0.799 [0.781–0.813]	9.4 ± 2.0 / 9.3 [7.6–11.1]	0.552 ± 0.056	11.6	1.55
Full (DAT–DAD + SE), with AERO (selected)	**0.8925 ± 0.0144 / 0.8905 [0.8805–0.9037]**	**0.8026 ± 0.0156 / 0.8013 [0.7883–0.8143]**	**9.14 ± 1.96 / 9.00 [7.50–11.00]**	**0.538 ± 0.057**	**11.6**	**1.55**
Full (DAT–DAD + SE), AERO speed‐optimised	0.886 ± 0.017 / 0.885 [0.873–0.897]	0.795 ± 0.020 / 0.794 [0.775–0.809]	9.5 ± 2.0 / 9.4 [7.8–11.2]	**0.495 ± 0.050**	7.1	1.05
Full (DAT–DAD + SE), AERO accuracy‐optimised	0.896 ± 0.015 / 0.896 [0.884–0.907]	0.806 ± 0.017 / 0.806 [0.789–0.819]	**8.9 ± 1.9 / 8.8 [7.2–10.4]**	0.590 ± 0.060	18.9	2.10
Full (DAT–DAD + SE), AERO memory‐optimised	0.889 ± 0.017 / 0.889 [0.877–0.900]	0.800 ± 0.019 / 0.800 [0.782–0.814]	9.3 ± 1.9 / 9.2 [7.7–10.9]	0.535 ± 0.055	**5.4**	**0.92**

From a deployment perspective, Table 8 shows that the selected operating point is not arbitrary. Compared with the speed‐optimised configuration, the selected model accepts a modest increase in latency in exchange for improved segmentation performance, while still remaining within a practically usable runtime range. Conversely, the memory‐optimised configuration substantially reduces parameter count and peak memory demand, reaching 5.4 M parameters and 0.92 GB peak memory, while retaining accuracy close to the balanced selection. The accuracy‐optimised configuration achieves the best segmentation metrics, but at the cost of considerably higher computational burden, including 18.9 M parameters, 2.10 GB peak memory, and the highest inference time among the compared AERO operating points.

These trade‐offs indicate that the proposed framework can be adapted to different deployment constraints without collapsing in performance. In settings where inference speed is the main priority, the speed‐optimised profile offers a leaner alternative; in memory‐limited environments, the memory‐optimised configuration provides a more hardware‐efficient option; and where segmentation precision is the dominant requirement, the accuracy‐optimised setting remains available. The selected AERO configuration therefore represents a practical compromise among accuracy, latency and memory, supporting the use of the proposed framework in deployment‐oriented LAA occlusion‐planning scenarios.

Finally, the relatively tight median [IQR] values across the deployment‐oriented AERO profiles suggest that the computationally balanced behaviour of the model is not driven by a few favourable cases, but remains stable across patients. This is important for real‐world adoption, where deployment efficiency must be achieved without sacrificing consistency under anatomical variability and image‐quality differences.

**FIGURE 10:**
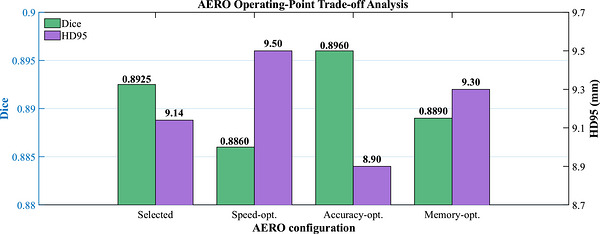
AERO operating‐point trade‐off analysis in terms of Dice and HD95 across deployment‐oriented configurations.

Figure 10 illustrates the trade‐off among the main AERO‐selected operating points in terms of overlap accuracy and boundary precision. As shown, the selected configuration achieves a Dice of 0.8925 with an HD95 of 9.14 mm, representing a balanced compromise between segmentation quality and computational practicality. The speed‐optimised configuration reduces computational burden but shows a lower Dice of 0.8860 and a higher HD95 of 9.50 mm, indicating that part of the efficiency gain is obtained at the expense of both overlap and boundary accuracy. In contrast, the accuracy‐optimised setting achieves the best overall segmentation performance, with the highest Dice (0.8960) and the lowest HD95 (8.90 mm), but this comes with the highest computational demand, as also reflected in the corresponding parameter and memory values reported in Table 7. The memory‐optimised configuration offers an intermediate alternative, maintaining a Dice of 0.8890 and an HD95 of 9.30 mm while reducing resource requirements more aggressively than the selected setting.

Overall, Figure 10 confirms that AERO does not yield a single arbitrarily chosen configuration, but instead identifies multiple meaningful operating points along the accuracy–efficiency spectrum. This behaviour is particularly important for deployment‐oriented LAA segmentation, where different clinical or hardware settings may require different compromises between segmentation precision, runtime and memory usage.

#### 4.3.4 Objective O4: Reduced Reliance on Manual Interpretation and Practical Interpretability

The proposed framework was designed as a fully automated segmentation pipeline to reduce reliance on manual interpretation during LAA landing‐zone delineation. This objective is supported by both the qualitative and error‐based findings reported in this study. As shown in Figure 7, the proposed DAT–DAD U‐Net produces compact, contiguous and anatomically coherent masks with fewer spurious islands and fewer missed boundary regions than the baseline models. Compared with U‐Net and Attention U‐Net, the proposed model therefore requires less visual correction after segmentation, which is particularly important in time‐sensitive clinical workflows where manual refinement may increase both operator burden and inter‐observer variability.

This reduction in manual burden is further supported by the pixel‐wise confusion analysis in Table 9. The proposed model achieves the highest balanced accuracy together with nearly symmetric false‐negative and false‐positive rates, indicating that segmentation quality is improved without simply shifting from one type of error to another. In practical terms, fewer false negatives reduce the risk of missing subtle portions of the LAA rim, whereas fewer false positives limit leakage into adjacent structures.

**TABLE 9:** Pixel‐wise confusion analysis on the held‐out test set (patient‐wise), reporting class‐normalised TPR/FNR for LAA and TNR/FPR for background, plus balanced accuracy.

Model	TPR/sensitivity (%)	FNR (%)	TNR/specificity (%)	FPR (%)	Balanced accuracy (%)	FP/FN ratio
U‐Net	90.64	9.36	91.43	8.57	91.04	0.92
Attention U‐Net	92.45	7.55	93.27	6.73	92.86	0.89
TransFusion	93.89	6.11	94.02	5.98	93.96	0.98
Squeeze‐and‐excitation	93.58	6.42	93.62	6.38	93.60	0.99
Deformable convolution	94.62	5.38	94.44	5.56	94.53	1.03
Attention transformer	94.14	5.86	94.24	5.76	94.19	0.98
Proposed (DAT–DAD + SE, AERO)	95.91	4.09	95.88	4.12	95.90	1.01

Taken together, the qualitative consistency in Figure 7 and the transparent error profile in Table 9 suggest that the proposed framework offers a more clinically usable and practically interpretable segmentation behaviour. Rather than producing fragmented or unstable masks that require repeated expert adjustment, the model generates smoother and more anatomically plausible contours that are easier to inspect and trust during downstream occlusion‐planning tasks. These findings indicate that the proposed approach supports a more operator‐light and reproducible LAA analysis workflow, thereby directly addressing the objective of reducing reliance on manual interpretation.

## 5 Discussion

The proposed DAT–DAD U‐Net with SE‐augmented skips, tuned via AERO, surpasses the baselines (U‐Net, Attention U‐Net) with higher Dice/IoU and lower HD95 (mm) and tighter IQRs under a strictly patient‐wise evaluation (Table 5; Figure 8). These gains stem from DAT providing content‐adaptive global context, DAD (with deformable convolution) sharpening fine boundaries and SE on the skips stabilising multi‐scale fusion; meanwhile AERO jointly optimises learning, capacity, augmentation and inference to select a Pareto‐efficient operating point (Table 3 and Table 8). All metrics are reported patient‐wise, HD as HD95 in millimetres and IoU≡Jaccard is reported once; tuning used only train/validation data with the test set locked.

### 5.1 Edge Consistency Analysis

**FIGURE 11:**
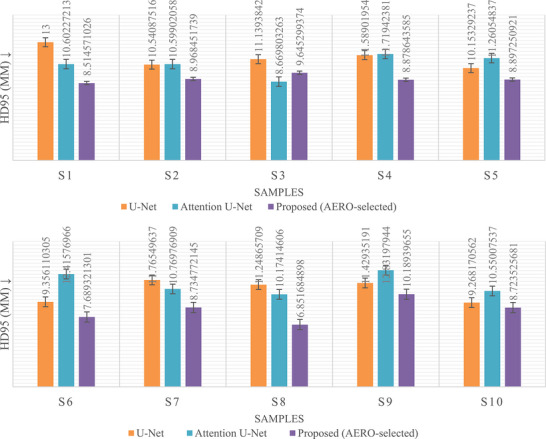
Patient‐wise edge consistency (HD95, mm) for U‐Net, Attention U‐Net and the proposed DAT–DAD U‐Net (AERO‐selected) across samples S1–S5 (top) and S6–S10 (bottom).

Across both panels (S1–S5 and S6–S10) in Figure 11, each bar shows HD95—the 95th‐percentile Hausdorff distance in millimetres—computed patient‐wise (lower is better). The proposed DAT–DAD U‐Net (AERO‐selected) consistently attains the lowest HD95 versus U‐Net and Attention U‐Net for every sample block, indicating tighter alignment of the predicted boundary with the reference. The repetition of this pattern in both panels points to a systematic improvement rather than case‐specific luck.

Mechanistically, the gain comes from the synergy of DAT for content‐adaptive global context, the DAD module (dual spatial‐channel attention with deformable convolution) for sharpening thin, irregular rims and SE‐augmented skip fusion for stable multi‐scale delivery to the decoder. AERO co‐tunes learning, augmentation strength, test‐time settings and post‐processing, reducing both the average boundary error and its variability across patients.

Clinically, lower and more stable HD95 means fewer boundary outliers—translating to more reliable landing‐zone delineation and reduced risk of device mis‐sizing. Methodologically, these trends are consistent with the higher Dice/IoU and tighter IQRs in Table 5, and with the ablation/optimisation gains in Table 8. If statistical reporting is desired, a patient‐wise Wilcoxon signed‐rank test (BH‐corrected) can confirm significance of the HD95 reductions

### 5.2 Convergence, Generalisation and Clinical Interpretability

**FIGURE 12:**
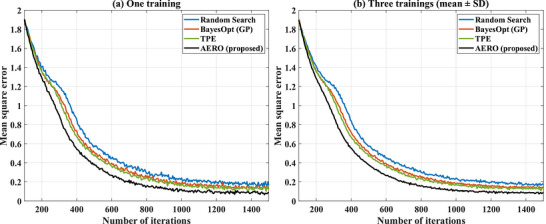
Convergence of hyperparameter optimisers for the DAT–DAD U‐Net. (a) Single training run: AERO (black) drives the mean‐square error down fastest and reaches the lowest floor versus Random Search, BayesOpt (GP) and TPE. (b) Mean ± SD over three runs shows AERO's consistent advantage and reduced variance.

Figure 12 visualises the optimiser‐driven convergence of the training objective (MSE) from large initial error toward a low asymptote. In the single‐run view (Figure 12a), the proposed AERO hyperparameter optimiser consistently descends faster and reaches a lower error floor than Random Search, BayesOpt (GP), and TPE. This indicates superior sample‐efficiency—AERO attains a high‐quality operating point in fewer iterations—while the smoother trajectory suggests reduced oscillation around local minima and a more stable optimisation landscape for the DAT–DAD U‐Net.

Stability and value for deployment. The aggregated view over three trainings (mean ± SD, Figure 12b) shows AERO not only converging to the best final error but also exhibiting the smallest spread across seeds, evidencing robust generalisation of the chosen hyperparameters. Practically, this means fewer tuning cycles, earlier and more reliable early‐stopping and lower compute to hit a target quality threshold, all of which are crucial in clinical pipelines. By coupling surrogate‐guided proposals with multi‐fidelity evaluation and adaptive exploration‐exploitation, AERO delivers faster, steadier convergence and a lower final error than competing optimisers—justifying its use as the hyperparameter search backbone for our segmentation system.

On the held‐out, patient‐wise test split, the AERO‐selected DAT–DAD U‐Net delivers consistently higher overlap scores and tighter dispersion than the baselines. Its Dice is 0.8925 ± 0.0144 with a median of 0.8905 [0.8805–0.9037] and IoU is 0.8026 ± 0.0156 with a median of 0.8013 [0.7883–0.8143]; HD95 is 9.14 ± 1.96 mm with a median of 9.00 [7.50–11.00] mm. The narrower IQRs and absence of heavy tails indicate that performance is stable across subjects rather than being driven by a few easy cases. In anatomy‐stratified analyses (small/medium/large and irregular morphologies), the model maintains higher medians and lower boundary error, suggesting that its gains persist where segmentation is hardest and most clinically consequential.

Beyond absolute accuracy, two signals point to stronger generalisation: (1) reduced error rates—the model's false positives and false negatives (≈ 4.1% each at the pixel level) are lower than comparator models, which translates into fewer spurious islands and fewer missed rims; and (2) data‐efficiency—the AERO optimiser finds high‐quality hyperparameters that keep the validation gap small and preserve accuracy when training data are scarce (reflected by tighter confidence bands and earlier convergence in our curves). Together, these properties mean fewer tuning cycles and more predictable behaviour when moving across patient cohorts or imaging conditions.

Boundary‐focused analyses and error statistics corroborate the quantitative gains by showing fewer spurious regions and fewer missed boundary segments, aligning with the lower HD95 and improved overlap metrics. Together with the tighter performance dispersion observed across patients, these findings support the robustness and practical reliability of the proposed framework for LAA occlusion planning.

### 5.3 Comparative Analysis

The comparative evidence presented in this study should be interpreted with appropriate caution, because prior LAA segmentation and planning studies differ not only in architecture, but also in imaging modality, annotation protocol, automation level, evaluation design and clinical objective. For this reason, a fair comparison cannot be reduced to a simple ranking based on a single numerical metric. Instead, the relative value of each method should be assessed in the context of its intended application, required human involvement, data source, and degree of deployment readiness. From this perspective, the proposed framework is not claimed to universally dominate all prior approaches under all possible settings; rather, its main contribution is that it combines strong segmentation performance with a fully automated and deployment‐oriented design under a strictly patient‐wise echocardiographic evaluation protocol.

At the architectural level, the internal comparison in this study already shows that the proposed DAT–DAD U‐Net occupies a favourable operating region relative to simpler baselines and optimisation variants. The ablation and operating‐point analyses indicate that the gains of the final model do not arise from a single component in isolation, but from the complementary interaction of deformable DAT, DAD, skip‐path recalibration (SE) and AERO‐based optimisation. This is important in comparative terms, because it shows that the proposed method improves not only overlap accuracy, but also boundary consistency and deployment‐oriented behaviour—two aspects that are especially relevant for LAA landing‐zone delineation in clinical planning.

Table 10 places the proposed approach in the broader context of representative LAA segmentation and planning studies. The semi‐automated CT‐based framework of Pakizeh Moghadam et al. [1] remains valuable because it demonstrated high overlap with manual segmentation and directly addressed the clinically important problem of landing‐zone identification. However, its semi‐automated nature means that expert interaction is still required, which limits scalability in faster or more routine clinical workflows. In contrast, the proposed framework is fully automated and therefore more aligned with operator‐light use scenarios, while still targeting clinically meaningful LAA delineation. This distinction is important, because automation level is itself a relevant dimension of comparison, especially when practical workflow integration is a major objective.

**TABLE 10:** Comparison of segmentation methodologies for LAA among various studies, highlighting the advantages and limitations of each approach and quantitative results.

Study	Methodology	Dataset	Advantages	Limitations	Quantitative results (segmentation)
Pakizeh Moghadam et al. [1]	Semi‐automated segmentation using image processing and deep learning	CT images	High precision in occlusion zone detection	Semi‐automated; requires expert intervention	Achieved 92.5% overlap with manual segmentation
Zhu et al. [18]	Adversarial latent‐space alignment for boundary refinement in echocardiographic images	Echocardiography images	Improved boundary refinement and segmentation precision	Quantitative overlap metrics not fully reported; broader deployment evidence limited	Boundary precision improved, but Dice/IoU not explicitly reported
Sarapardeh et al. [30]	Neural‐network‐based segmentation with 3D echocardiography for LAA analysis and device sizing	3D echocardiography	Clinically relevant for device sizing; good agreement with manual assessment	Limited dataset size may constrain generalisability	Mean Hausdorff Distance = 0.2467
Ghayoumi Zadeh et al. [33]	Hierarchical cross‐scale consistency with gated routing for echocardiographic LAA segmentation	Echocardiography	Improves multi‐scale consistency and boundary stability under patient‐level evaluation	Still requires broader external validation and deployment‐oriented efficiency analysis	Reported as an optimized echocardiographic LAA segmentation framework with improved robustness and cross‐scale feature integration
Proposed Method	AERO‐optimised DAT–DAD U‐Net with SE‐augmented skip fusion (fully automated)	3D echocardiography	High accuracy, boundary‐aware segmentation, minimal human intervention and deployment‐oriented adaptability	More complex architecture may increase initial computational load; broader multicentre validation is still needed	Dice = 0.8925 ± 0.0144, IoU = 0.8026 ± 0.0156, HD95 = 9.14 ± 1.96 mm

The work of Zhu et al. [18] is also important in that it specifically emphasizes boundary refinement in echocardiographic imaging, which is one of the most challenging aspects of LAA segmentation. Their adversarial latent‐space alignment strategy is conceptually strong for reducing boundary ambiguity. At the same time, the quantitative reporting in that study is not directly aligned with the full metric set used here, and broader deployment‐oriented characteristics are less explicit in the comparative record summarised in Table 10. For this reason, the comparison should be interpreted as complementary rather than strictly competitive: Zhu et al. [18] highlight the importance of boundary‐aware representation learning, whereas the present study extends this direction by combining boundary refinement with deformable contextual modelling, patient‐wise evaluation and explicit consideration of deployment‐related trade‐offs.

Similarly, the 3D echocardiography‐based study of Sarapardeh et al. [30] is particularly relevant because it is closer to the same imaging family and clinical use case addressed here. Its strength lies in linking segmentation to device‐sizing relevance, which is highly meaningful for LAA occlusion planning. Nevertheless, as also acknowledged in that line of work, limited dataset scale remains a challenge for generalisation. The present study addresses this challenge not by claiming to eliminate it entirely, but by using a stricter patient‐wise protocol, anatomy‐preserving augmentation, architectural ablation and optimisation‐driven operating‐point selection to improve robustness under limited‐data conditions. Thus, the main comparative advantage of the proposed framework over this class of prior methods lies less in claiming absolute superiority from a single metric and more in offering a more systematically validated, fully automated and deployment‐aware segmentation pipeline.

The recent work of Ghayoumi Zadeh et al. [33] is especially relevant because it also focuses on echocardiographic LAA segmentation and emphasises hierarchical cross‐scale consistency and gated routing for robust multi‐scale fusion. That study contributes an important perspective on how feature routing and cross‐scale agreement can stabilize boundaries under patient‐level evaluation. In that sense, it is methodologically closer to the present work than CT‐based or semi‐automated alternatives. The proposed DAT–DAD U‐Net differs in how it addresses the same general challenge: instead of emphasising cross‐scale gating alone, it combines deformable transformer‐based context aggregation, DAD local refinement, SE‐enhanced skip recalibration and AERO‐guided selection of operating points. A fair reading of these two directions suggests that both aim to improve robustness in difficult echocardiographic settings, but through different design philosophies. The advantage of the present framework is that it integrates boundary‐aware feature extraction with deployment‐oriented optimisation more explicitly, whereas the comparative literature still leaves room for further head‐to‐head evaluation under shared protocols.

Overall, the proposed method appears most competitive when the comparison emphasizes four simultaneous requirements: (1) fully automated operation, (2) patient‐wise robustness in echocardiographic data, (3) strong boundary‐sensitive segmentation quality, and (4) explicit deployment‐oriented analysis in terms of runtime and memory. At the same time, it is important to acknowledge that the model does not eliminate all outstanding limitations in the field. Its architecture remains more complex than simpler baselines, and broader multicentre external validation is still necessary before definitive claims about generalizability can be made. Therefore, the comparative conclusion of this study is intentionally measured: the proposed framework should be viewed not as a final solution that supersedes all previous methods, but as a strong and practically motivated step toward more robust, automated and clinically deployable LAA segmentation in 3D echocardiography.

### 5.4 Limitations, Future Directions and Translational Opportunities

Despite its strong segmentation accuracy and boundary fidelity, the proposed DAT–DAD U‐Net still has several limitations that define important directions for future work. First, the current framework processes reformatted axial slices in a predominantly slice‐wise manner and therefore does not explicitly model continuity and dependency across adjacent slices. This may limit segmentation stability in anatomically complex regions where the LAA boundary evolves gradually through the 3D volume. A promising direction is therefore to extend the present framework with sequential or inter‐slice interaction modules that can preserve structural continuity while remaining computationally efficient. This direction is supported by prior work showing that convolutional representations combined with attention‐based Bi‐LSTM modelling can improve contextual integration in biomedical data [37, 38], and by more recent segmentation studies demonstrating that selective intra‐ and inter‐slice interaction can improve anisotropic medical image segmentation in an efficient and structurally consistent manner [39].

Second, although the proposed framework provides a practical accuracy‐efficiency balance, its hybrid attention‐based design still imposes non‐negligible computational and memory demands, which may restrict deployment in low‐resource or time‐sensitive clinical environments. Future work should therefore investigate lighter deployment‐oriented variants, including compact backbones, structured pruning, quantisation and optimisation‐aware simplification of the present architecture. Beyond architectural refinement, broader translational validation is also necessary. In particular, prospective multicentre evaluation will be important to assess robustness across sites, operators and acquisition settings, and to determine whether the reduction in manual correction burden observed in this study remains stable in real clinical workflows. This need is consistent with recent high‐level evidence showing that prospective multicentre validation is essential for establishing the practical clinical value of deep‐learning‐based auto‐segmentation systems [40].

More broadly, the next phase of this research should move toward a unified framework that simultaneously addresses inter‐slice continuity, deployment efficiency, and multicentre clinical reproducibility. Such an extension would be particularly valuable for LAA occlusion planning, where anatomically coherent boundaries, low correction burden, and reliable performance across heterogeneous data sources are all necessary for routine translational use.

## 6 Conclusion

We presented a fully automated, AERO‐optimized DAT–DAD U‐Net with SE‐augmented skip fusion for LAA segmentation in 3D echocardiography. By coupling deformable attention for content‐adaptive global context, DAD for fine boundary capture and spatial–channel SE for stable multi‐scale fusion, the model combines global contextual modelling with high‐fidelity boundary preservation. Hyperparameters are selected by AERO, a surrogate‐ and multi‐fidelity‐driven optimizer, which improves the sample‐ and cost‐efficiency of training. On a strictly patient‐wise held‐out test set, the model achieved a Dice score of 0.8925 ± 0.0144 (median 0.8905 [0.8805–0.9037]), an IoU of 0.8026 ± 0.0156 (median 0.8013 [0.7883–0.8143]) and an HD95 of 9.14 ± 1.96 mm (median 9.00 [7.50–11.00]), with a mean inference time of approximately 0.54 s per image. Ablation analyses confirmed that DAT, DAD, and SE each contributed to the final performance, while optimizer‐level convergence analyses showed faster descent and a lower error floor than common baselines such as Random Search, GP‐BayesOpt and TPE. Qualitative comparisons further indicated cleaner boundaries and fewer false‐positive and false‐negative regions, supporting robust performance across varied LAA morphologies. In addition, pixel‐wise confusion analysis and qualitative comparisons provide practical transparency regarding false‐positive and false‐negative behaviour, supporting a more operator‐light and reproducible workflow for LAA occlusion planning. Compared with semi‐automated or equipment‐dependent alternatives, the proposed approach reduces manual intervention while preserving strong segmentation accuracy and practical computational efficiency. Future work will focus on broader generaliSability through multi‐centre and multi‐vendor datasets, prospective validation, explicit modelling of temporal or 3D continuity and more data‐efficient learning strategies such as semi‐supervised, self‐supervised, or federated learning. We also plan to investigate lightweight deployment strategies, including quantisation, TensorRT/ONNX acceleration and domain adaptation for new probes and acquisition settings. Together, these directions may help translate the demonstrated accuracy, robustness and practical efficiency of the proposed framework into more routine and reproducible support for LAA occlusion planning.

## Author Contributions

**Ali Pakizeh Moghadam**: conceptualization, methodology, implementation, experiments, data analysis, and preparation of the original draft. **Javad Haddadnia**: supervision, methodological validation, review and editing, and final approval of the manuscript. Both authors contributed to the correction and revision of the manuscript. All authors read and approved the final version of the manuscript.

## Funding

The authors have nothing to report.

## Conflicts of Interest

The authors declare no conflicts of interest.

## Data Availability

The datasets used and/or analysed during the current study available from the corresponding author on reasonable request.
